# Ethical Attitudes toward COVID-19 Passports: Evidences from Spain

**DOI:** 10.3390/ijerph182413098

**Published:** 2021-12-11

**Authors:** Mario Arias-Oliva, Jorge Pelegrín-Borondo, Ala Ali Almahameed, Jorge de Andrés-Sánchez

**Affiliations:** 1 Management and Marketing Department, Complutense University of Madrid, 28040 Madrid, Spain; mario.arias@ucm.es; 2 Department of Business Management, Social & Business Research Laboratory, Universitat Rovira i Virgili, 43002 Tarragona, Spain; 3 Economics and Business Department, University of La Rioja, 26006 Logroño, Spain; jorge.pelegrin@unirioja.es; 4 Social and Business Research Laboratory, Universitat Rovira i Virgili, 43002 Tarragona, Spain; a.mahameed82@gmail.com

**Keywords:** ethical judgment, immunity passport, COVID-19 passport, COVID-19, attitude, Multidimensional Ethics Scale (MES)

## Abstract

A so-called COVID-19 passport or Immunity passport (IP) has been proposed to facilitate the mobility of individuals while the SARS-CoV-2 pandemic persists. A COVID-19 passport can play a key role in the control of the pandemic, specifically in areas with a high density of population, and the help of smart city technology could be very useful to successfully implement IPs. This research studies the impact of ethical judgments on user attitudes toward using vaccine passports based on a Multidimensional Ethics Scale (MES) that contains five ethical constructs: moral equity, relativism, egoism, utilitarianism, and contractualism. Regression analysis shows that MES satisfactorily explains attitude (R^2^ = 87.82%, *p* < 0.001) and that a positive evaluation in moral equity, egoism and utilitarianism is significant (*p* < 0.001). The objective of the passport (variable leisure) shows a significant negative moderating effect on moral equity (coefficient = −0.147, *p* = 0.0302) and a positive one on relativism (coefficient = 0.158, *p* = 0.0287). Adjustment by means of fsQCA shows that five ethical constructs satisfactorily explain both favorable and unfavorable attitudes toward IPs. Solutions explaining acceptance attain an overall consistency (cons) = 0.871 and coverage (cov) = 0.980. In the case of resistance, we found that cons = 0.979 and cov = 0.775. However, that influence is asymmetrical. To have a positive attitude toward the passport, it is a sufficient condition to attain a positive evaluation on a single ethical factor. On the other hand, when explaining resistance, and with the exception of the recipe ~utilitarianism (cons = 0.911 and cov = 0.859), explanatory prime implications require the interaction of at least two variables. Likewise, the context in which the passport is required is significant to explain rejection.

## 1. Introduction

COVID-19 has transformed the way we work, interact and live. Based on the global statistics on COVID-19 and up to the beginning of October 2021, the number of infections reached more than 235 million, of which around 4.8 million deaths and 212 million cases of recovery from the disease have been recorded [1]. Our everyday life changed dramatically, especially during the peak of the infection weaves. At the very beginning of the pandemic, only traditional measures used for centuries were initially adopted to cope with the situation: social distancing. 

Maintaining a greater than usual physical distance or quarantines were used in the 14th century in Venice during the Black Death [2]. Many countries used extreme measures, such as lockdowns, to avoid the spread of the pandemic, such as Spain, the UK, and France [3]. These effective and ancient measures have an enormous impact on both social life and economic activity. 

This pandemic has caused millions of workers to lose their jobs across the world and was the largest decline for the quarterly Gross Domestic Product (GDP) since the Great Depression, which took place between 1929 and 1932 [4]. For instance, the International Air Transport Association (IATA) reported a drop in international passenger demand by 75.6% below the 2019 demand [5]. In the USA, the GDP reported the first annual decline for 2020 (3.5%) since the financial crisis in 2007, and this is considered to be the lowest drop since 1946 [6]. In Europe, the unemployment rate increased by 7.3% for January 2021 compared to the same period in 2020, which was 6.6% [7].

COVID-19 testing was an innovative strategy to lift confinement restrictions while waiting for a cure or effective vaccine [8]. On 9 November 2020, Pfizer and BioNTech made history by announcing that their coronavirus vaccine had an efficacy rate of over 90% [9]. Since this moment, vaccines have become one of the most effective solutions to overcome the pandemic and its consequences.

The combination of COVID-19 testing and vaccines create a new way to fight against the pandemic: immunity certificates. Immunity passports (IP) are defined as potential tools for recording and sharing the immunity status of an individual [10]. These immunity certificates have been named in many different ways, including as a Digital Green Certificate [7], Vaccine passport [11], and COVID-19 passport [12]. They represent a digital or physical document that certifies an individual is immune to SARS-CoV-2. This could be because a person has been vaccinated against COVID-19, received a negative test result, or recovered from COVID-19 [13].

Restricting the mobility of individuals has been considered as an effective way to minimize and control the spread of COVID-19. These restrictions could promote social distancing, partial or complete lockdowns, closing public transportations and borders, or working from home [14]. A study proved that the lockdown that was imposed in the Chinese city of Wuhan reduced the outbreak of COVID-19 in the entire country by almost 58.7% [15]. Another study found that the number of new COVID-19 cases in Italy was related directly to the mobility of individuals across the country [16]. 

They claimed that if travel between cities across the country is reduced, the total number of new cases will decrease. Moreover, the impact of mobility on COVID-19 transmission was studied for 52 countries by using data from Apple and Google [17]. Their results showed that mobility is correlated with the intensity of COVID-19 transmission. Findings showed that, for 73% of analyzed countries, the reduction in mobility reduced the transmission of COVID-19. The relationship between global mobility and the COVID-19 pandemic outbreak was proven [18]. 

Some countries in Europe, such as Spain, consider the IP as a useful and effective tool to resume mobility safely, which will be reflected positively in the country’s economy. Austria, Bulgaria, Greece, Malta, and Slovakia joined Spain in a joint declaration to promote the IP as soon as possible [19]. For Chinese citizens who are traveling overseas, the vaccination status and COVID-19 test results can be shown on the Chinese social media apps WeChat, which was launched on March 8, 2021. In the same context, the IATA justified the need for the IP, which will offer accurate information on passengers’ health status regarding COVID-19 [20].

On the other hand, and from the individual perspective, not everyone supports the use of IPs. In fact, different countries and international institutions are opposing this proposal from an ethical perspective, considering potential problems, such as discrimination, data privacy, and freedom of movement [21]. For instance, vaccination and COVID-19 test results could be classified under personal health data, meaning that processing shall be prohibited as per Article 9 of the General Data Protection Regulation (GDPR). The exception could be related to the public interest, such as controlling the COVID-19 outbreak [22]. However, the uncertainty of vaccine ability to prevent virus transmission should be considered. 

Potential discrimination between the people who already have been vaccinated, and the people who have not been vaccinated yet could represent a way of classifying people based on their COVID-19 situation. The freedom of movement is another concern that could become a problematic issue, especially for people who would not be able to take the vaccine because of health constraints. This would imply that individuals not vaccinated yet will not be able to go on international travel or may suffer domestic and local mobility restrictions [23]. 

Moreover, how much time the immunity will last for vaccinated people is still unknown, and the scientists are following up with individuals who have received the vaccine to learn if their immune response is durable over time [24]. Another factor that should be considered is the financial consequences associated with the need to do the COVID-19 test frequently for those who are not vaccinated. The timeline that the world will need in order to ensure that vaccination reaches all countries is another critical concern, where expectations indicate that, by the end of 2021, the majority of adults in developed countries will be vaccinated [25]. 

However, most African countries will not be able to offer the vaccine to their population until 2023. The previous issues taken together make it difficult to obtain this passport for all individuals, at least before the completion of vaccine distribution to the entire world, which likely will not happen before 2023 at best. This could reinforce the ethical concerns regarding the ability of everyone to obtain the IP. 

Measures against COVID-19, such as wearing a mask, isolation and of course, the implementation of IPs, have deep ethical implications. Regarding IPs, there is much literature reflecting on these concerns as demonstrated above. Likewise, there is a great deal of empirical assessments about the influence of sociodemographic factors, such as gender, age or educational level, on the acceptance of health measures against SARS-CoV-2, such as, vaccination (see the survey [26]). However, to the best of our knowledge, there is not much empirical research on the impact of ethical aspects about the perception of measures against SARS-CoV-2. 

This research attempts to fill this gap by considering the ethical perceptions that act as keystones in stimulating attitudes toward immunity passports. To answer this question, we use a Multidimensional Ethics Scale (MES) to analyze how its dimensions (moral equity, relativism, egoism, utilitarianism, and contractualism) influence people’s attitudes toward the implementation of a vaccine passport. MES has been used to determine the influence of ethical judgment in several contexts [27]. However, this focus has not been used to evaluate attitude toward health measures with fair moral concerns, such as to prevent COVID-19, and therefore we can consider that our topic is a novel focus in public health literature. 

## 2. Literature Review

### 2.1. Ethical Considerations of Immunity Passports

A study about scientists’ opinions about IPs found a lack of common agreement toward the ethical issues of the proposed COVID-19 passports [28]. The main focus was on their design and implementation, given the importance and relevance implications of passports as a result of the increased availability of vaccines and their efficacy.

The idea from the IPs is to reduce mobility restrictions and control people’s movement. Using paper documents as vaccine certificates and test results could lead to fraud and forgery of documents. Therefore, most countries and international institutions are proposing IPs as a mobile digital application, which will include the individuals’ COVID-19 health status, if they have received the vaccine or not, and may contain other information, such as travel history and people locations. 

This could expose this information to a serious privacy risk [23,29]. In this context and from a technological perspective, new solutions could cause challenges to societies in the form of ethical concerns. Apart from than privacy and manipulation concerns, this could entail restricting the mobility of people who are poor, who do not have access to the technology, who cannot take the vaccine because of health issues, or cannot pay for COVID-19 tests. Governments in the future could use the same method to discriminate between people based on their immunity [30].

Grouping people based on their COVID-19 health status is another ethical concern. This could cause potential harm to minority groups that are not vaccinated. In addition to that, the availability of vaccines and testing could be an issue at the individual and country level, as the pace of vaccination in developed countries is faster than in developing ones, and COVID-19 testing access could be easier for wealthy people. This fact represents another ethical issue induced by the unfair access to vaccines and testing [23].

### 2.2. The Influence of Ethical Judgment on Attitude

The first step of ethical awareness is to be conscious that an issue may have ethical implications. We may consider a specific action as an ethical issue if harming or benefiting others as the result of performing this action freely [31]. Then, the ethical judgment will be used to evaluate action that is associated with the ethical issue. Ethical judgment could be defined as “a cognitive process in which an individual is to judge which course of action is morally right’’ [32] (p. 445). As a result, ethical judgment will be established by considering the priority for actions that are morally right over other considerations [33].

To make ethical judgments, individuals may use more than rationale, since multiple dimensions are required to understand the actual meaning of ethical judgment [34]. The Multidimensional Ethics Scale (MES) has been used to measure ethical judgments and includes moral equity, relativism, utilitarianism, egoism, and contractualism. These five philosophical constructs are generally accepted to explain why an individual may do the right thing [35]. A definition of each dimension is as follows:

1. Moral equity, which is considered a strong predictor of attitude toward usage for specific situations, can be defined as human perception of what is wrong and right, in addition to justice and fairness [33,36]. It is based on the justice theory [37].
2. Relativism considers that individuals’ actions are based on parameters/guidelines, which are established in the social/cultural system. It refers to the “perception of what is right versus wrong based on guidelines embedded in the social/cultural system, rather than individual considerations” [33] (p. 629). There is no common rule that can be applied to everyone because the normative beliefs are a function of individuals or culture, which represents the basic concept of this dimension [34].
3. Utilitarianism refers to “an action based on cost and benefits analyses, such that the action will bring about the greatest good for the greatest number” [33] (p. 628). It is based on teleological or consequentialist theories and considers that individuals will compare one action to another. As a result, utilitarianism promotes efficiency. In other words, the more efficient actions may produce more utility than less efficient actions and are, therefore, more ethical [34]. In the public health field, whereas [37] justified a moral obligation to be vaccinated from an utilitarianism point of view, Clarkson and Jasper [38] observed that people who judged positively regarding vaccination from this perspective stated a better attitude toward mandatory vaccination.
4. Egoism, which is based on teleological or consequentialist theories, refers to considering only consequences to the individuals rather than utilitarianism, which is considering repercussion to society [34]. This means the individual will behave to promote the self-interests, such as self-promotion and satisfaction [33]. In this way, the self-promotion of individuals can influence their attitude and intention to behave ethically in case there are benefits for these individuals as a result of their decision [27,36].
5. Contractualism is a dimension that includes the concepts of rules, obligations, contracts, and duties [34]. This is considered a deontological dimension and proposes that a contract exists between business and society to represent a base of individual perception of what is considered right versus what is considered wrong [33]. It entails unspoken and unwritten contracts that exist between individuals and their society, and these contracts influence all behaviors [36]. Giubilini et al. [37] provided arguments from the contractualism perspective supporting that vaccination is an ethical duty.

Attitude refers to people’s general evaluation of a specific object, such as products and brands. This evaluation may include feelings toward that object, beliefs about it, and intentions toward it, which could all impact the person’s choices and actions [39]. In this context, a negative ethical judgment could involve a negative attitude. Contrariwise, a positive attitude could be stimulated by a positive ethical judgment [40]. 

Several studies have analyzed the influence of MES scale dimensions on consumer attitudes and intentions to use context. For instance, one study applied the MES scale to investigate the ethical judgments and behavioral intentions of Information and Communications Technology (ICT) college students in three scenarios: the action is not perceived as a violation of contract or illegal, the action is perceived as harmless, and the violation of privacy as a result of the action [41]. 

The results proved that there were no significant differences among the three scenarios regarding ethical judgments. The students showed that they were less likely to be involved in the third scenario action (the violation of privacy) than in the first or the second one. Regarding the relation between ethical judgment and behavioral intention, moral equity for the second scenario and contractualism for the third scenario showed a significant impact on behavioral intention on the peer level. However, relativism showed a significant impact for three scenarios on the individual level, and moral equity showed a significant impact on the behavioral intention for the second and third scenarios on the individual level. 

Another study developed multiple scenarios to analyze individuals’ perceptions of different ethical situations that are involved in C2C e-commerce practices, studying the factors that may impact attitudes and behavior intentions [36]. The authors targeted undergraduate students. Students were asked to evaluate different scenarios to determine their belief about how ethical behavior should be and to define the proposed predictors of their decision. The authors offered different items to students in order to examine the impact of ethical dimensions on behavioral intention in an e-commerce setting. 

The results confirmed the full impact of moral equity and egoism on behavioral intention, partial support of relativism and contractualism, and no significant impact of utilitarianism on behavioral intention. In the same context, the MES scale was used to determine student attitude and behavior while using technology [42]. The participants were asked to answer items that were used to investigate the impact of ethical dimensions on behavioral intention on both individual and peer levels. 

The results indicated that relativism showed a significant impact on behavioral intention on the peer level, and egoism, moral equity, and contractualism had a significant impact on behavioral intention on the individual level. However, utilitarianism did not show a significant impact on the behavioral intention for both the individual and peer levels. Another study analyzed the impact of ethical judgment on the decision to become a cyborg by applying the MES scale on intention to use insideables [27]. 

The authors developed a survey and administrated it to university students from Chile, Denmark, Mexico, Japan, China, Spain, and the USA with an average of 1563 participants. The research results supported a positive significant relationship between intention to use insideables and moral equity, egoism, relativism, and utilitarianism. Egoism showed the most explanatory power, followed by moral equity, utilitarianism, and relativism. 

The impact of situational factors has been widely investigated in the field of consumer’s behavior. Thus, [43] showed the influence of situational factors (i.e., per-antecedent states, temporal perspective, and lifestyle changes) on online shopping adoption. The results confirmed the positive relationship between situational factors and online shopping adoption. 

In the healthcare context, the moderating effect of situational factor has been confirmed on m-health acceptance [44], the impact of situational factors on selection of health information technology selection [45], and role of situational factors on individuals’ acceptance to share their healthcare information on electronic platforms [46]. As far as we are concerned, IP may be used in three different situations: international travel [47], domestic mobility [48], and public and private facilities access [10]. This research evaluates the use of the IP for travel and leisure regulation. These two situational factors may moderate the ethical judgment–attitude relationship. 

Despite empirical research on individual factors influencing attitude toward vaccination [26] and other preventive measures against COVID-19 [49] is so wide, this does not follow in the case of COVID-19 passports. At this regard, in a sample from US, [50] did not find relevant habitual sociodemographic factors, such as income, age or cultural status to explain opinion on IP. However, [50] also reported that females are more reluctant toward COVID-19 passport. On the other hand, [49] found similar results in Spain. 

However, in that paper, it was noticed that women are slightly more favorable than men to IP and also that being vaccinated is the most individual influencing factor on attitude toward COVID-19 passport. In this paper, we are not discussing how sociodemographic factors influence attitude that, on the other hand, have not shown a clear influence on attitude on COVID-19 passport [49,50]. We are strictly interested in ethical judgements systematized by a MES and the settings in which IP may be implemented. Therefore, we consider as explanatory variables the five ethical constructs exposed above and the moderating context in which IP will be mandatory.

Thus, we propose testing the following hypotheses based on the aforementioned considerations:

**Hypothesis** **1** **(H1).**  *Moral equity in favor of an Immunity Passport positively affects the Attitude toward it.*

**Hypothesis** **2(H2).**  *Relativism in favor of an Immunity Passport positively affects the Attitude toward it.*

**Hypothesis** **3(H3).**  *Egoism in favor of an Immunity Passport positively affects the Attitude toward it.*

**Hypothesis** **4(H4).**  *Utilitarianism in favor of an Immunity Passport positively affects the Attitude toward it.*

**Hypothesis** **5(H5).**  *Contractualism in favor of an Immunity Passport positively affects the Attitude toward it.*

**Hypothesis** **6(H6).**  *Situational Factors affect the relation between Ethical Judgment and Attitude toward Immunity Passports.*

**Figure 1:**
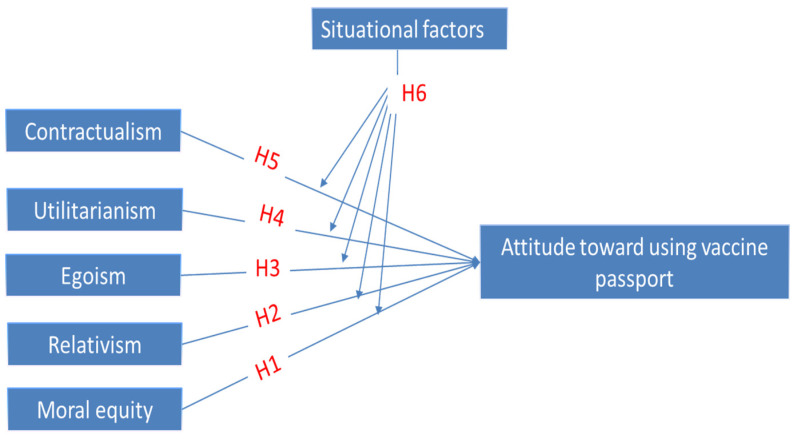
The proposed model.

Figure 1 is showing our proposed model, which is summarizing the relation between MES scale dimensions and attitude toward the IP.

## 3. Materials and Methods

### 3.1. Materials

In order to verify the hypotheses, we conducted a survey of residents of Spain over 18 years of age. For the survey, gender quotas were established (50% men and 50% women) and age quotas (18–24 years = 25%; 25–44 years = 25%; 45–64 years = 25%; plus 64 years = 25%). The interview period coincided with news about the COVID-19 passport (16 April 2021 to 29 April 2021). Before starting the questions, the survey displayed a brief explanation about IPs:

“*COVID-19 certificates or passports are currently being developed: these are documents that allow certifying that a person cannot spread COVID-19 to others, because they are vaccinated, have medical proof that shows that they are not infected, or have had COVID-19 and therefore is immune*.”

The sample obtained consisted of 400 surveys (200 from women and 200 from men; 100 from people between 18 and 24 years old, 100 from people between 25 and 44 years old, 100 from people between 45 and 64 years old, and 100 from old people 35 or more) obtained in the area of Madrid. Of these 400 people in the sample, 21.3% had received at least one vaccine for COVID-19, and on average 1.83 tests (PCR, antigens, or others) had been carried out to detect COVID-19. 

After stating that 400 may be a correct and reachable number of answers, we accepted, in all cases, the first answers that covered the quotas of age and gender that were previously fixed. Likewise, we only considered as a valid survey that completely answered. Notice that it is a cross sectional study that was carried on at the beginning of the public debate on immunity passports (April, 2021). 

The percentage of people with one shot of vaccine in the sample is according with that existing in Spain in that data. Thus, we can outline that population is representative of great urban areas of Spain, such as Madrid, Barcelona or Valencia, and also representative of Spanish vaccination situation in that data.

Trained interviewers located people of these ages and gender. This was conducted through digital means (e-mail, Facebook, etc.) or by phone. They supplied the interviewed people a Google Form link that only allowed one response per electronical device. The questionnaire was answered without the interviewer supervision but, on the other hand, answered persons have an interviewer of reference if any concerns arose. The questionnaire did not demand any data that could identify surveyed person as, for example, name or passport number. 

Thus, the anonymity of the collected data was ensured. All participants were people of legal age. No permission was obtained from a board or committee ethics approval; it was not required as per applicable institutional and national guidelines and regulations. Voluntary completion of the questionnaire was taken as consent for the data to be used in research, informed consent of the participants was implied through survey completion. As we carry on the survey, it was not possible to determinate whether a contacted person that received the survey link actually answered the questions. 

The Composite MES (Composite Multidimensional Ethics Scale) by [35] has been used to measure ethical judgment toward the mandatory use of the Covid passport. This scale is an extension of the original MES scale that was developed by [34]. Three of the main theories of ethical judgment are included in the original scale, but elements related to egoism and utilitarianism were missing [51] (p. 290). 

In the Composite MES scale, [35] (p. 663) added items referring to these dimensions of ethical judgment, in such a way that they included items from the five main theories of ethical judgment [35] (p. 650): relativism, morality, contractualism, egoism, and utilitarianism. This MES scale and the Composite MES scale are frequently used scales in the literature to measure people’s ethical judgment [27,52,53]. In the scale measurement, a semantic differential scale −5 to +5 was used.

The items on this scale include Unjust/Just; Unfair/Fair; Not morally right/Morally right; Not acceptable to my family/Acceptable to my family; Culturally unacceptable/Culturally acceptable; Traditionally unacceptable/Traditionally acceptable; Personally useless/Personally useful; Not personally satisfying for me/Personally satisfying for me; Produces the least social utility/Produces the greatest social utility; Minimize benefits and maximize hurt/Maximize benefits and minimize hurt; Violates an unwritten contract/Does not violate an unwritten contract; and Violates an unspoken promise/Does not violate an unspoken promise.

The scale [54] has been adapted to measure the attitude of having to use the COVID-19 passport on a mandatory basis to travel outside the country, between cities, or within their city; to use COVID-19 passport on a mandatory basis to enjoy leisure activities, such as concerts or restaurants. In the measurement of the scale, the semantic differential scale −5 to +5 has been used. Scale items include: Bad/good; Foolish/wise; Ineffective/effective; and Negative/positive.

### 3.2. Methods

As a first step, we check the internal consistency of scales by using usual measures: Cronbach’s alfa (CA), composite reliability (CR), Dijkstra–Henseler measure (ρA), and average variance extracted (AVE). Likewise, we run factorial exploratory analysis to check the existence of additional dimensions in scales. 

In the second step, we fit a principal component linear regression on Attitude (ATT), which is explained by moral equity (ME), relativism (RE), egoism (EG), utilitarianism (UT), and contractualism (CONT). All variables are quantified by the standardized punctuation obtained in factorial analysis, i.e., in fact, we are estimating a principal component regression. As we are also interested in measuring and testing the significance of moderating effects due to passport context (travel vs. social relationship), we introduce a dichotomous variable LEISURE that takes 1 if the judgment comes from a leisure perspective and 0 in the case of considering a traveling objective that interacts with all ethical factors. Thus, the linear regression model fitted is:

ATT = a_0_ + a_1_ × ME + a_2_ × REL + a_3_ × EGO + a_4_ × UT + a_5_ × CONT +a_6_ × ME × LEISURE + a_7_ × REL × LEISURE +a_8_ × EGO × LEISURE +a_9_ × UT × LEISURE +a_10_ × CONT × LEISURE (1)

where, coefficients a_1_, a_2_…a_5_ measure the influence of each ethical construct on attitude, and a_6_, a_7_…a_10_ fits the differential effect on the attitude of each kind of ethical judgment when it comes from a social interaction perspective. Therefore, model (1) enables us to test not only hypotheses 1–5, but also 6.

In the third step, we perform a fuzzy set qualitative comparative analysis (fsQCA) with the same input and output variables. To build up their membership functions, we normalize the factorial punctuations of ATT and ethical variables within [0, 1]. On the other hand, LEISURE again takes values within {0, 1}. 

Fuzzy set qualitative comparative analysis is an interesting complement to regression analysis as it has shown in practical cases by [55,56] within a business management setting since it focuses on the analysis of input and output variables in a different manner. Whereas regression methods only find the best solution to explain an output, fsQCA provides a set of combinations of input variables, so-called recipes, which can explain the output. 

Likewise, regression methods measure the mean effect of input variables on output by means of their coefficients and, on the contrary, fsQCA identifies the combinatorial interactions between variables to produce a given output [57]. Likewise, the significance of regression coefficients is evaluated with Student’s t and the overall goodness by the regression model with the coefficient of determination (R^2^). On the other hand, in a fsQCA context, we have similar measures to evaluate any recipe. Consistency (cons) of a solution plays a similar role than t-ratio. 

The coverage (cov) of a solution measures the capability of the recipe to embed all observations and so it can be assimilated to R^2^ [58]. Notice that linear regression also assumes linear symmetrical relation between variables. This fact does not follow with fsQCA [57]: the determinants of producing a positive attitude are not necessarily symmetrical with those that produce a negative judgment on attitude. 

Whereas in business research using conjointly correlational and fsQCA methods is common, this does not follow in public health literature and consequently in revised literature about people’s attitude toward SARS-CoV-2 measures. All revised papers ground their analyses in correlational methods. Thus, the use of fsQCA supposes a novelty in empirical public health studies. As far as our analysis is concerned, fsQCA will allow discovering combinatorial and asymmetrical effects of input variables on personal perceptions about the mandatory use of IP.

Thus, our fsQCA adjustment implies evaluating ATT, but also, its negation ~ATT where “~” stands in fsQCA notation “negation”. Thus, the following two Boolean functions are fitted:

ATT = f (ME, REL, EGO, UT, CONT, LEISURE)(2a)

~ATT= f (ME, REL, EGO, UT, CONT, LEISURE)(2b)

Estimates of (2a) and (2b) are obtained by using fsQCA 3.1. by [59]. That software offers three solutions: complex, parsimonious and intermediate. By using Quine-McCluskey algorithm fsQCA 3.1 fits essential prime implicates. This prime implicates conform so-called Qualitative Comparative Analysis (QCA) complex solution (CQA-CS) and parsimonious solution (CQA-PS). CQA-CS is usually hard to interpret and is build up with no more assumption than data. 

The simple solution is done by using any remainder over the non-observed configuration of variables in order to make the solution as easy as possible regardless of whether it constitutes an “easy” or “difficult counterfactual” case [59]. Thus, following [60], fsQCA 3.1 can also use simplifying assumptions that are a theory-driven hypothesis of how a given condition might be causally related to the outcome. It must be a hypothesis in non-observed configurations if an input variable contributes to output exclusively when present, absent, or in both cases. These counterfactuals allow obtaining a third solution, a so-called QCA intermediate solution (QCA-IS).

A complex solution does not introduce any counterfactual in the analysis, and theoretically this solution must be exclusively used to interpret data. Unfortunately, many times the recipes contained in that solution are hard to understand. That is why [60] suggests using QCA-IS since it supposes a compromise between QCA-PS and QCA-CS and [57] suggest combining QCA-PS and QCA-IS to state core conditions (those that are in QCA-PS and so in QCA-IS) and peripheral conditions, i.e., conditions that are only in QCA-IS.

The fourth step is testing the predicting capability of fsQCA methodology. To do this, we follow the procedure in [57]. We split answers into a subsample for fsQCA estimation and a sample to test the extrapolation capability of that fsQCA estimation. In our case, the estimation (holdout) sample were 350 (50) answers that were chosen randomly.

## 4. Results

Table 1 shows the statistical descriptive of the items and their standardized loadings. In all the cases, these loadings are >0.7. Table 2 suggests that scales internal consistency exists since CA, CR and ρA are >0.7 and AVE > 0 in all the cases.

Regarding regression model (1), we observe the following patterns (Table 3):

- ME, EG, and UT have a clear positive relation with ATT (*p* < 0.001). Thus, H1, H3, and H4 are accepted.
- RE has a positive relation with ATT at a certain statistical significance (*p* = 0.0516). Thus, H2 can be accepted but this acceptance is weaker than H1, H3, and H4.
- CONT has a negative non-significant link with ATT.
- Adjusted R^2^ is above 87%, and F statistic shows that the overall model has statistical significance (*p* < 0.001).
- Likewise, coefficients linked with moderating effects of passport context show that it influences the effect of ME and REL on ATT. However, its impact on other ethical constructs is rejected. ME importance over ATT is decreased when the motive of COVID passport is leisure activities (*p* = 0.0302). The importance of REL on ATT is increased when the passport is used for those leisure activities (*p* = 0.0287). Thus, H6 can be accepted for ME and REL but not for other ethical variables.

Results of the QCA-IS of (2a) and (2b) are shown in Table 4. When explaining ATT (2a), QCA-IS is very straightforward to interpret. A positive judgment in any assessed ethical dimension causes a positive attitude toward passport with cons > 0.9 and cov > 0.85 in all the cases. Likewise, the dichotomous variable LEISURE does not appear in any recipe, so, that variable has neither influence nor moderating capability in attitude. 

For ~ATT, we can identify the following patterns:

- All recipes present a consistency clearly above 0.8, i.e., they explain a part of negative attitudes toward a passport.
- We hypothesized, the variables ME, REL, EG, and UT come negated in all the explanatory combinations of ~ATT. Thus, we can conclude that H1, H2, H3, and H4 can be accepted.
- On the other hand, when fitting ~ATT, CONT appears, as we expected, negated in two recipes (~REL*~CONT with cons = 0.86 and cov = 0.44 and ~ME*~CONT*~LEISURE with cons = 0.82, cov = 0.45) but in a recipe comes affirmed (~EG*CONT, cons = 0.86, cov= 0.44). This last recipe suggests that there is a significant opinion stream that rejects IP despite judging positively it from a contractualism perspective due to egoist reasons. However, contractualism in that configuration acts as a peripheral. Thus, these findings suggest that could not be a defined sign between ~ATT and CONT and so, H5 could be rejected.
- We can show that contextual variable LEISURE moderates some others to induce a negative attitude. Thus, the recipe ~EG*~LEISURE (cons = 0.84, cov = 0.44) indicates that a negative judgment on EG must be accompanied by a mobility objective to produce a negative perception. A negative attitude is linked exclusively with a travel objective in the recipe ~ME*~CONT*~LEISURE (cons = 0.822, cov = 0.45). On the other hand, the combination ~REL*LEISURE (cons = 0.84, cov = 0.47) suggests that also relativism is moderated by the contextual use of COVID passport. Notice that whereas that LEISURE in the condition ~EG*~LEISURE has a peripheral importance, in ~ME*~CONT*~LEISURE and ~REL*LEISURE has a full presence.

Table 5 shows the results of the assessment on fsQCA prediction capability. We can observe the following patterns:

- With the estimation subsample, we obtain the same configurations for ATT as those of whole sample (compare Table 4 and Table 5). We can also observe that recipes from estimation subsample attain similar consistency and coverage in holdout subsample.
- As far as ~ATT is concerned, by using the estimation subsample we do not obtain exactly the same recipes as with whole subsample (compare Table 4 and Table 5). However, this fact is not an exception as it can be checked in the example [57] and likewise this does not validate fsQCA prediction capability. still can obtain similar configurations for ATT as those of whole sample (compare Table 4 and Table 5). As in the case of ~ATT that recipes from estimation sample attain similar cons and cov in testing sample.

## 5. Conclusions

An immunity passport (IP), COVID-19 passport or Vaccine Passport has been proposed by different countries and international organizations to facilitate the mobility of individuals at the local and international levels or to grant access to different activities, such as leisure (concerts, restaurants, etc.). By using this passport, people who have been vaccinated or have negative test results of the virus will be exempted from the restrictive measures that are imposed by governments [61]. 

In order to minimize people’s selfishness in relation to that measure, we feel that, in addition to mandatory regulations, it is necessary to provide persuasive and clear information showing that the benefits of immunity passports are clearly greater than the problems that they could allegedly cause regarding personal freedom and privacy. In this context, the European Commission presented their proposal for the digital green pass on the 17th of March 2021, which will be used to prove that the holders have a negative test or have received the vaccine, and it will become available in all EU countries by the 1st of July 2021. It will be available as a mobile app and as a paper document with a QR code, free of charge, in national and English languages, and it will be valid in all EU countries [7]. 

On the other hand, not everyone is supporting the proposition of passports, and certain countries and international institutions are opposing this proposal from an ethical perspective, considering potential problems, such as discrimination, data privacy and freedom of movement [21]. In this context, this research has studied the impact of ethical judgment on user attitudess toward the IP by using MES dimensions. Few studies have discussed the ethical implications of using the IP. Neither the attitude toward COVID-19 passports nor the impact of ethical aspects on the attitude has been investigated yet.

This research used two quantitative methods to analyze the survey: linear regression and an fsQCA. These methods produced complementary results. Regression model (1) detected a significant positive relation of moral equity, relativism, egoism, and utilitarianism with attitude but a negative non-significant influence of contractualism. Likewise, we detected a moderating capability by the objective of the passport over ME and REL but not on other ethical dimensions. 

The use of fsQCA aided us in confirming a clear positive relation of ME, REL, EG, and UT with ATT, and thus, undoubtedly H1, H2, H3, and H4 can be accepted. A positive judgment on the contractualism dimension causes a positive attitude (see recipe CONT for ATT). However, it may cause ~ATT (the recipe ~EG*CONT). Thus, H5 can be accepted neither from a regression perspective nor from fsQCA results. Regarding H6, we checked that context where the passport must be used does not influence positive attitudes; however, this fact does not follow for a negative perception. In that case, there are recipes where ME, REL, EG, and CONT are moderated with the dichotomous variable LEISURE. 

FsQCA is very suitable to study phenomena where the impact of input variables is completely asymmetrical over the presence and absence of a given output. That is the case of the variables producing success and failure in complex organizations. In this regard, Woodside [62], in his fourth tenet, indicated that causes of organization’s failure and success must be completely different, i.e., recipes indicating the negation of the outcome (i.e., failure) must be unique and not the mirror opposites of recipes of its affirmation (success). 

However, fsQCA can be used to state combinatorial interactions of input factors to produce an output when there exists a completely symmetrical impact on affirmation and negation of the outcome [57]. Notice that our application is not that in [63] but is the acceptance and resistance of IP. Our results indicate that ethical factors impact vaccine passports asymmetrically, but that asymmetry is not complete. There exists a symmetrical recipe for the acceptance and rejection of IP (utilitarianism causes acceptance and ~utilitarianism causes rejection); however, the mainstream of prime implications of ~ATT (seven) are not the mirror opposites of recipes fitted for ATT.

As we exposed above, there is a little empirical research on the impact of ethical perceptions on attitudes toward measures against COVID-19. However, we can point out that our findings are in the same line to those in [38] that reported relevant positive perceptions from the utilitarianism point of view into opinions on mandatory vaccination. Likewise, our empirical results suggest that arguments from the utilitarianism ethical perspective exposed by Giubilini et al. [37] in a vaccination setting can be used to justify the implementation of IP. However, this does not follow for their contractualist arguments.

Our paper has several implications from theoretical point of view. We demonstrated that ethical perceptions measured by means of an MES can satisfactorily explain attitudes toward public health measures when they embed moral concerns. We applied this framework to the analysis on IP, but our focus can be extended to any other measure, e.g., mandatory vaccination. 

Likewise, the combination of statistical methods and fsQCA allows for a more complete analysis of evaluated variables in the perception of a studied health measure. Whereas correlational methods allow stating the average influence of an assessed variable on the acceptance of a studied measure, fsQCA discovers combinatorial effects of input factors on attitudes toward evaluated health measures as well as asymmetrical impacts of explanatory variables on the acceptance and resistance behaviors toward a given public health policy.

We feel that this paper provides valuable findings for public health decision makers. Perceptions on ethical dimensions quantified in a MES are relevant to induce a positive judgement on the use of IP. Thus, for an effective implementation of an immunity passport, health authorities must convince citizens that it is fair (moral equity), is required by close persons, such as peers and family (relativism), supposes a positive measure to attain personal objectives (egoism), provides a social utility (utilitarianism) and is required from the contractualism dimension. Likewise, whereas to have a positive perception is sufficient for a favorable judgement in a solely one ethical dimension, resistance to IP is produced by the combination of negative perceptions in at least two factors and/or moderated by the context where IP is going to be mandatory.

One of the ethical issues related to COVID-19 passports is the availability of vaccines and testing. For instance, the pace of vaccination in developed countries is faster than in developing ones, and COVID-19 testing access could be easier for wealthy people than vulnerable and poor people, which may create a question about moral equity in terms of fairness, justice, and the right of everyone to have access to the vaccine and/or COVID-19 testing [23]. From this premise, the research results confirmed the significant impact of moral equity on attitude toward IPs. According to that, decision-makers should ensure the availability of vaccines and COVID-19 testing to everyone before enacting the mandatory use of passports. 

The ethical judgment could be a result of social and/or cultural tendencies, which could support or disagree with the use of COVID-19 passports. For instance, not everyone is supporting the proposition of IPs; certain countries and international institutions are opposing these passports. They have doubts about ethical issues that could be associated with using passports, such as discrimination, data privacy, and mobility rights [21]. 

In the same context, vaccination and COVID-19 test results could be classified under personal health data, and its processing is prohibited as per Article 9 of the General Data Protection Regulation (GDPR). The exception could be related to the public interest, such as controlling the COVID-19 outbreak [22]. Consequently, the research results support the importance of ethical judgment in terms of the relativism dimension, which showed a significant impact on user attitudes toward IPs and in both directions. 

Some countries have taken incentive procedures to encourage citizens and residents of their territories to take the vaccine, as it represents the fastest and safest solution to this pandemic thus far. Some European countries are allowing vaccinated travelers who are permitted to visit Europe without the need for a 14 day quarantine [63], whereas a COVID-19 passport has been proposed to facilitate the mobility of individuals at the local and international levels by exempting people who have been vaccinated or have negative test results of the virus from the restrictive measures that are imposed by governments [61]. 

From this perspective, promoting the benefits of using passports to facilitate user mobility could be required to stimulate positive attitude toward these passports. This is aligned with our research findings, which confirmed that attitude toward IP is affected by user egoism, which is related to the benefits that will be associated with individual decisions or actions. On the other hand, utilitarianism, which is considered as consequences to society rather than to the individual as a basis of ethical judgment, could impact the use of COVID-19 passports at the society level. 

For instance, the idea of using the vaccine passport is to reduce mobility restrictions and control people’s movement, which could be reflected positively in the local and international economies, including airlines, restaurants, travel agencies, and global trade activities [10]. This may justify the importance of ethical dimensions “utilitarianism” on user attitudes toward vaccine passports in both directions: positively or negatively, upon user perceptions, for instance, of the greatest or least social utility that is associated with using these passports.

Even though the research results confirmed the impact of ethical judgments on attitudes toward IPs and the moderating effect of travel objectives, future research is required to apply the same in different countries. Future studies need to investigate user attitudes toward passports when we have a better understanding of the effectiveness of immunity and its duration for former patients and vaccinated people. Furthermore, user perceptions of the benefits and/or drawbacks of using COVID-19 passports could be changed after the actual use of them.

A possible limitation of the study is that it collected a not relatively high number of responses (400) at a very specific moment in time (April 2021), in which the COVID-19 certificate had not yet been implemented in a significant demographical area of Spain, including Madrid. However, the debate on the ethical implication of its use was (and still is) a burning topic. Obtaining more precise conclusions about how the acceptance of the IP will evolve would require a set of longitudinal surveys covering, for example, at the time when this document was written, when the format of the IP was already known, there were already experiences on their requirement in travel and access to public places, the proportion of vaccinated population was around 80%, and COVID-19 vaccines were fully available. 

The results would be different in another Spanish geographical area (for example, a rural region) or country with different sensitivity toward the danger posed by COVID and/or with a longer expectation of vaccination coverage.

## Figures and Tables

**Table 1:** Descriptive of items on the survey and factor loadings.

	Traveling Purpose			Leisure Purpose		
	Mean	Std. Dev.	Loading	Mean	Std. Dev.	Loading
Attitude 1	5.97	3.43	0.904	5.78	3.63	0.929
Attitude 2	5.95	3.40	0.907	5.74	3.54	0.939
Attitude 3	6.17	3.23	0.872	5.88	3.40	0.928
Attitude 4	6.20	3.21	0.821	6.05	3.41	0.868
Moral equity 1	4.97	3.42	0.877	4.91	3.48	0.934
Moral equity 2	5.21	3.31	0.901	5.02	3.38	0.951
Moral equity 3	4.91	3.43	0.884	4.91	3.51	0.934
Moral equity 4	5.24	3.35	0.898	5.09	3.38	0.921
Relativism 1	6.14	3.40	0.85	5.82	3.44	0.868
Relativism 2	5.55	3.33	0.895	5.29	3.33	0.92
Relativism 3	5.36	3.10	0.852	5.11	3.26	0.902
Egoism 1	6.12	3.45	0.942	5.78	3.59	0.94
Egoism 2	5.72	3.47	0.942	5.49	3.56	0.94
Utilitarianism 1	6.39	3.35	0.925	6.02	3.46	0.944
Utilitarianism 2	6.42	3.12	0.925	6.03	3.31	0.944
Contractualism 1	5.16	3.37	0.964	5.09	3.41	0.955
Contractualism 2	5.27	3.27	0.964	5.14	3.31	0.955

**Table 2:** Measures on the internal consistency of scales.

	Traveling Purpose				Leisure Purpose			
	CA	ρA	CR	AVE	CA	ρA	CR	AVE
Attitude	0.953	0.953	0.953	0.834	0.969	0.970	0.969	0.888
Moral equity	0.949	0.949	0.949	0.861	0.970	0.970	0.970	0.914
Relativism	0.922	0.924	0.923	0.799	0.942	0.945	0.942	0.845
Egoism	0.938	0.939	0.938	0.884	0.936	0.936	0.936	0.880
Utilitarianism	0.919	0.920	0.920	0.851	0.941	0.941	0.941	0.888
Contractualism	0.962	0.963	0.962	0.928	0.953	0.953	0.953	0.910

**Table 3:** Results of the regression (1).

Variable	Coefficient	t-Ratio	*p*-Value
moral equity	0.368	8.82	<0.001
relativism	0.095	1.95	0.0516
egoism	0.200	5.09	<0.001
utilitarianism	0.370	9.19	<0.001
contractualism	−0.053	−1.46	0.1455
moral equity× leisure	−0.142	−2.17	0.0302
relativism× leisure	0.158	2.19	0.0287
egoism× leisure	−0.030	−0.50	0.6164
utilitarianism× leisure	−0.051	0.89	0.3763
contractualism× leisure	−0.027	−0.50	0.6193
R2 = 87.82% F statistic = 577.28 (*p* < 0.001)			

**Table 4:** fsQCA findings for ATT = f(ME, REL, EG, UT, CONT, SR) and ~ATT = f(ME, REL, EG, UT, CONT, and SR).

		ATT = f(ME, REL, EG, UT, CONT, LEISURE)									
Configuration		1		2		3		4		5	
moral equity		●									
relativism				●							
egoism						●					
utilitarianism								●			
contractualism										●	
leisure											
consistency		0.972		0.951		0.937		0.912		0.941	
coverage		0.814		0.879		0.900		0.946		0.803	
Solution consistency = 0.871 Solution coverage = 0.980											
	**~ATT = f(ME, REL, EG, UT, CONT, LEISURE)**										
Configuration	1	2	3		4		5	6	7		8
moral equity					**⊗**		**⊗**				**⊗**
relativism			**⊗**				**⊗**		**⊗**		
egoism		**⊗**			**⊗**			**⊗**			
utilitarianism	**⊗**										
contractualism			⊗					•			**⊗**
leisure		⊗							●		**⊗**
consistency	0.911	0.843	0.860		0.899		0.864	0.862	0.840		0.822
coverage	0.859	0.448	0.884		0.888		0.917	0.436	0.469		0.451
consistency = 0.979 coverage = 0.775											

Note: Big circle (●) indicates presence of a condition and circles with x (⊗) its absence. Large circle is for core conditions, small circles for peripheral condition and blank space, “don’t care” condition.

**Table 5:** Results when testing the prediction capability of fsQCA models (QCA-IS solutions).

	ATT = f(ME, REL, EG, UT, CONT, LEISURE)			
	Estimation sample		Test sample	
Configuration	cov	cons	cov	cons
moral equity	0.806	0.973	0.859	0.969
relativism	0.876	0.949	0.9	0.961
egoism	0.897	0.939	0.921	0.924
utilitarianism	0.944	0.909	0.954	0.934
contractualism	0.803	0.939	0.799	0.955
coverage = 0.977				
consistency = 0.864				
	**~ATT = f(ME, REL, EG, UT, CONT, LEISURE)**			
	**Estimation sample**		**Test sample**	
Configuration	cov	cons	cov	cons
~utilitarianism	0.858	0.912	0.728	0.934
~egoism*~leisure	0.447	0.846	0.45	0.821
~relativism*~contractualism	0.883	0.864	0.893	0.832
~moral equity*~ relativism	0.918	0.863	0.909	0.878
~egoism*contractualism	0.437	0.869	0.442	0.777
~relativism*leisure	0.473	0.845	0.439	0.794
~moral equity*~ contractualism*leisure	0.462	0.825	0.422	0.777
coverage = 0.975				
consistency = 0.779				

Note: “*” stands for the Boolean product

## Data Availability

Data is available by demanding it from any of the authors.
